# The Role of School Nurses in Promoting Positive Sexual Health Education: A Scoping Review

**DOI:** 10.1177/10598405251337769

**Published:** 2025-05-13

**Authors:** Bianca Nymo, Sara Berg Eriksen, Anne-Martha Utne Øygarden

**Affiliations:** 1 Department of Nursing and Health Promotion, 60499 Oslo Metropolitan University, Oslo, Norway

**Keywords:** adolescents, children, public health nurse, school nurse, positive sexuality, sexual education, comprehensive sex education, sexual and reproductive health and rights, sexual reproductive education, scoping review

## Abstract

Media reports indicate that young people are discontented with current sexual education approaches, feeling that current practice disproportionately emphasizes negative aspects like sexually transmitted infections and unwanted pregnancies. A scoping review was conducted using Arksey and O'Malley's framework to examine how school nurses contribute to fostering positive sexuality through sexual education in schools. Out of 406 unique articles screened, 17 met the inclusion criteria. The majority were from the USA and England. The findings highlight the significant contributions of school nurses in sexual education programs. However, it also indicates a need for broader representation, particularly from Nordic countries. A more diverse perspective could enrich understanding and practices in promoting sexual health among youth. The findings also emphasize the preference of youth for external, qualified educators such as school nurses to deliver sexual education. The study calls for more research to enhance sexual health promotion among youth.

## Introduction

Research shows that sexual education leads to better and more positive attitudes toward reproductive and sexual health (UNESCO et al., 2018). The World Health Organization (WHO) defines sexual education as a lifelong process in which individuals acquire knowledge, skills, attitudes, and values related to sexuality and their sexual and reproductive health, with the aim of preserving their well-being (WHO, 2023). Sexual education encompasses sexual development and reproductive health, body image, human relationships, intimacy, emotions, and gender roles. Education should be tailored to age, knowledge, and cultural background (WHO, 2023) and should be rooted in scientific information, positively charged, and encourage critical thinking and sexual well-being.

Sexual health is seen as mutually affecting overall health, describing positive attitudes, feelings about one's own body, and knowledge as crucial factors in building a secure sexual identity and positive behavioral patterns (Norwegian Ministry of Health and Care Services, 2017–2022, p. 8). Internationally, guidelines exist where sexuality is viewed in connection with physical, emotional, and cognitive health and as a natural part of life (UNESCO et al., 2018). Comprehensive sexual education is presented as a valuable tool for promoting sexual well-being. The guidelines highlight the promotion of the positive aspects of sexuality and the importance of fostering healthy, respectful attitudes and values (UNESCO et al., 2018).

The current generation of youth actively engages with social media, television, and newspapers to highlight deficiencies in current sexual education, considering it an important source of information and knowledge. They desire a more comprehensive curriculum with an increased focus on pleasure. Adolescents perceive current education as characterized by negativity, with a primary focus on sexually transmitted diseases and unwanted pregnancy (Mathisen & Salvesen, 2023).

UNESCO highlights comprehensive sexual education as a tool for promoting good sexual health among children and adolescents (UNESCO et al., 2018). Comprehensive sexual education provides knowledge, skills, attitudes, and values about sexual health based on research and human rights. While comprehensive sexual education is not a standardized program, it serves as guidance for the development of international curricula. Internationally, the role of the school nurses is practiced differently. In Norway and Sweden, the role of school nurses is a statutory service (Sveriges riksdag, 2010; The Norwegian Directorate of Health, 2017), but to our knowledge, it is not a statutory obligation in other countries. The content and framework of sexual education vary widely and are influenced by numerous factors such as educators, children, and adolescents’ participation in education, attitudes and perceptions of sexuality, and cultural, political, and economic conditions (UNESCO et al., 2018; WHO, 2023). Sexual education in the United States varies despite the development of a comprehensive guide for sexual health education in schools. Through defined learning objectives and recommendations, the National Sex Education Standards seeks to improve the quality and consistency of sexual health education in the United States (Future of Sex Education Initiative, 2020). The majority of states provide guidelines. However, the final decisions regarding the timing and methodology of teaching are made by individual school districts. This leads to inconsistencies, meaning a student's access to comprehensive sexual education depends largely on their school and district. We see a similar trend in Norway, where sexual education is shaped by the institutional policies of the school and the pedagogical approaches of the educators, resulting in variations in both content and quality (Future of Sex Education Initiative, 2020; The Norwegian Directorate of Health, 2016).

### Purpose of the Review

To our knowledge, there are no reviews that outline sex education's contribution to positive sexual attitudes and behaviors. Therefore, this scoping review of sex education research pertaining to sexual education focuses on the role of school nurses in promoting positive sexual health among children and adolescents through sexual education. The review aims to answer the question: What does the literature say about how school nurses can promote positive sexuality among children and adolescents through sexual education in schools?

## Method

A scoping review provides an overview of a topic without assessing the quality of the literature, with the intention of informing practice, research, and guidelines (Levac et al., 2010; Tricco et al., 2018). Arksey and O'Malley describe four main reasons for choosing a scoping review as a method: to examine the breadth and content of the existing literature, assess the need to conduct a systematic review, gather and disseminate research findings, and identify gaps in existing literature (Arksey & ÓMalley, 2005).

The authors conducted a literature study and used a scoping review as a method to cover a wide breadth of literature on the topic of promoting positive sexuality through sexual education. The five-step framework developed by Arksey & O'Malley, further refined by Levac et al., was employed for structure, along with PRISMA-ScR for reporting the results (Arksey & ÓMalley, 2005; Levac et al., 2010; Tricco et al., 2018). The five steps in the framework are (1) identifying the research question, (2) identifying research, (3) study selection, (4) charting the data, and (5) collating, summarizing, and reporting the findings (Arksey & ÓMalley, 2005).

### Identification of the Research Question

The existing literature is extensively scoped and read before determining the research question. The research question and a priori methodology were specified in a protocol (Nymo & Eriksen, 2024). The research question should be broad, while also defining the target population, context, and outcomes (Levac et al., 2010).

### Identification of Relevant Research

The search strategy was developed by the first and second authors with guidance from a search specialist. From December 2023 to February 2024, we conducted a systematic search in five databases: Epistemonikos, CINAHL, EMBASE, ERIC & MEDLINE. The search strategy was:

*barn* OR *unge* OR *ungdom* OR (*adolescents* or *teenagers* or *young adults* or *teen* or *youth*) OR (*children* or *adolescents* or *youth* or *child* or *teenager*) OR *helsesykepleier* OR (*public health nurse* or *community health nurse* or *district nurse*) OR (*school health nurse* or *school nursing* or *school nurse*) OR (*school health services* and *adolescent health* and *health education* and *self-care*) OR *child health nurse* AND *seksualundervisning* OR (*sexual education* or *sex education* or *sex ed*) OR *sexual health education* AND *seksualundervisningsprogram* OR *seksualundervisningsopplegg* OR *comprehensive sex education* OR *comprehensive sexual education* OR sexual education program* OR sex education program* AND *positiv seksualitet OR trygg seksualitet OR sexual health OR sexual health promotion OR sexual behaviours* OR *(attitudes or perceptions)* OR *holdninger*

A timeframe of 10 years was set due to the awareness of previous research on sexual education provided to children and adolescents, which has largely focused on preventing harmful sexual behavior.

### Study Selection

The relevant studies are selected based on predetermined inclusion and exclusion criteria. We included all types of literature, encompassing academic papers, scientific literature, research articles, books, guidelines, news articles, and social media content about sexual education programs and the promotion of positive sexuality involving a population of youth and children from 0 to 18 years in a school setting. Studies had to be published between the years 2013 and 2024 and written in English or a Scandinavian language. These are the languages mastered by the author team and there were no funds available for study translations. All countries were included. We excluded studies delivered outside of the educational context and studies involving parents and sexual education for adults and the elderly.

All records identified in the searches were imported into EndNote, and duplicates were deleted. References were imported into Rayyan systematic review software (Ouzzani et al., 2016), a webtool designed to help researchers working on scoping reviews and other knowledge syntheses. Using Rayyan, the authors independently screened all titles and abstracts for relevance against the inclusion and exclusion criteria. The authors promoted all abstracts they considered relevant to full-text screening. Having obtained the publications in full text, the authors assessed their relevance against the inclusion criteria. Studies that met all eligibility criteria were included. At both screening levels, discrepancies or difficulties were deliberated, and consensus was reached by discussion.

### Data Extraction

The data extraction process involves applying a common analytical framework to all the included research reports (Arksey & ÓMalley, 2005). Using a data extraction sheet, the two main authors extracted information, which was checked for accuracy and completeness. The following data were extracted from all publications: the author, year of publication, country, design/method, context, number of participants, study population, and findings. In accordance with the scoping review methodology, a methodological quality assessment of the included studies was not performed (Arksey & ÓMalley, 2005).

### Collating, Summarizing, and Reporting the Results

The last step in the Arksey and ÓMalley (2005) framework entails summarizing the study and presenting findings and results. Descriptive and thematic analysis of the data was utilized (Arksey & O’Malley, 2005). Data findings are presented through both textual descriptions and tabular representations, employing narrative presentations of the results. Analysis and coding of our findings were conducted individually and reviewed collectively, resulting in a final count of 43 codes. These 43 codes were then grouped under five main themes.

## Findings

**Figure 1.**
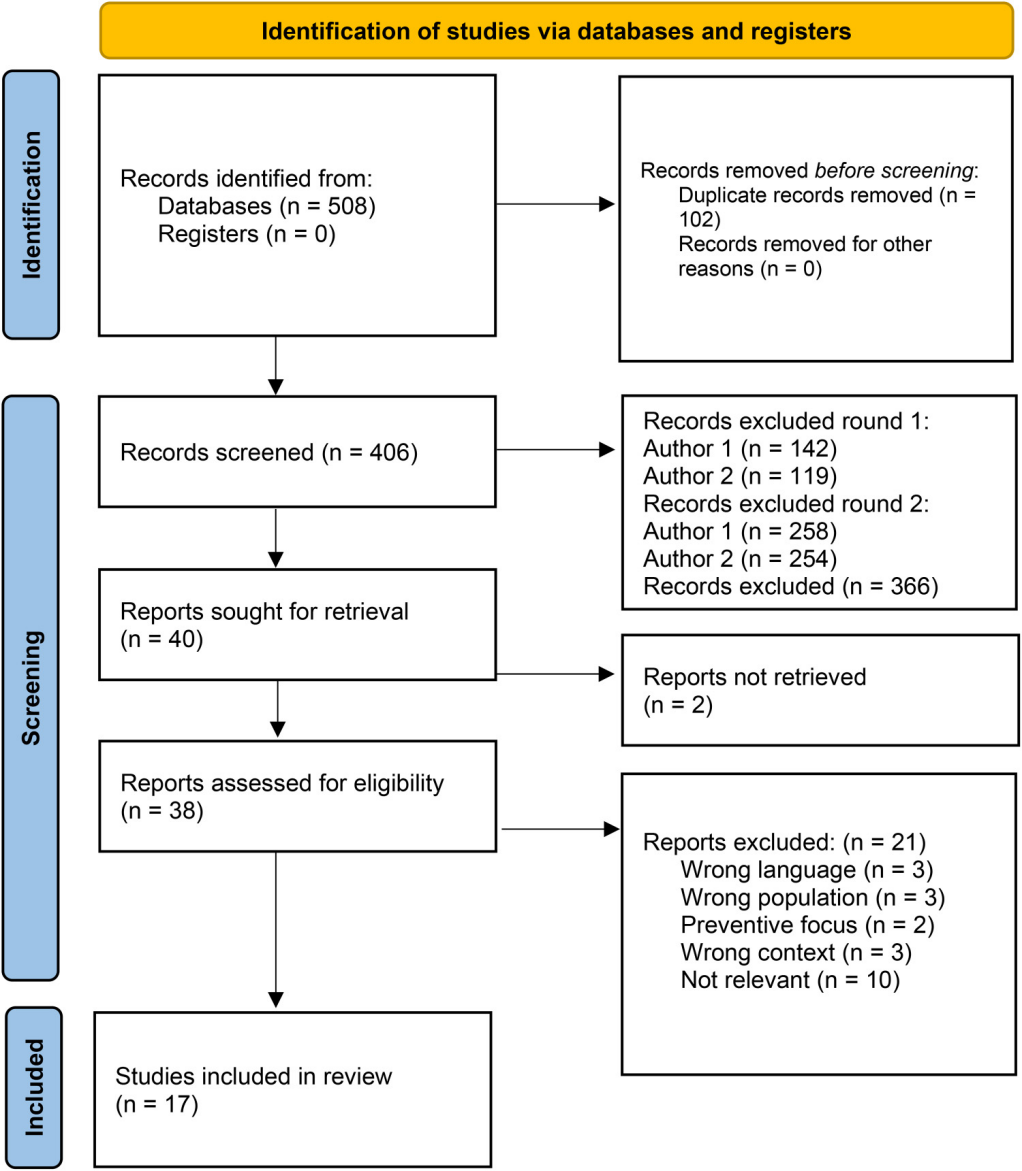
PRISMA flow diagram for article selection (Tricco et al., 2018).

From all five database searches after duplicate check, 406 unique articles were obtained, of which 40 were included for full-text reading, and 366 were excluded. A total of 38 articles were read in full text, as we could not access the full text for two articles. All publications read in full text were in English. We included 17 articles based on the inclusion and exclusion criteria. We have chosen to use the term sexual education throughout this study despite slightly different terms being used in the different studies. The study selection procedure is shown in the PRISMA flow diagram (Tricco et al., 2018) (Figure 1).

The mapping yielded 17 articles from database searches across 10 countries (Table 1). Most articles originated from the United States and England (52%), with notably limited findings from the Nordic region (6%) (Silivri et al., 2021). The sample sizes ranged from 14 to 24,579 participants, with a total of 32,272 children and adolescents and 32 school nurses. Three studies (Corcoran et al., 2019; Ericksen & Weed, 2023; Seiler-Ramadas et al., 2021) and two journal commentaries (Feuchtwang, 2016; Lepkowska, 2016) did not provide the number of study participants and are therefore not included in the calculations. One study (Ericksen & Weed, 2023) shares the same study population as another (Goldfarb & Lieberman, 2021). Findings indicate increased research on the topic, with nine of 17 (53%) studies conducted since 2020.

**Table 1.** Summary Characteristics of the Included Studies (*N* = 17).

Characteristics	Studies *N* (%)
Year of publication	
2013–2019	8 (47%)
2020–2023	9 (53%)
Country	
Austria	1 (6%)
Cyprus	1 (6%)
England	3 (17%)
Indonesia	1 (6%)
Nepal	1 (6%)
Netherlands	1 (6%)
Sweden	1 (6%)
Taiwan	1 (6%)
Tanzania	1 (6%)
USA	6 (35%)
Populations	
School nurses	32 (1%)
Students	32,272 (99%)
Number of participants^a^	
<50	2 (12%)
50–2000	7 (41%)
2001–25,000	2 (12%)
Literature studies	6 (35%)

*Note.* ^a^ Six studies were literature studies and did not include a number of participants.

We grouped results under five main themes: (1) youth participation, (2) the role of educators, (3) sexual education frameworks, (4) shift in perspectives on sexuality, and (5) expanding teaching topics within sexual health. Table 2 gives an overview of the included studies (Table 2), while Table 3 presents an overview of the included studies and coding title (Table 3).

**Table 2.** Overview of Included Studies.

Authors (year)	Title	Country	Design and method	Study population and context	Findings	Codes
Acharya et al. (2018)	Nepalese School Students Views About Sexual Health Knowledge and Understanding	Nepal	Qualitative Study Utilizing Eight Focus Group Interviews: Thematic Analysis	*N* = 78 students High schools in Nepal.	Despite sexual education being recognized as important for promoting good sexual and reproductive health among adolescents, the study reveals an ongoing needs for improving the approach to teaching. The involvement of adolescents in the educational process and the adoption of relevant pedagogical methods should be developed to enhance students’ knowledge and understanding of their sexual health.	1, 2, 3, 4, 5
Aranda et al. (2018)	Listening for commissioning: A participatory study exploring young peoplés experiences, views, and preferences of school-based sexual health and school nursing	England	Qualitative focus group interviews	*N* = 74 students/15 focus group	This study explores the youth perspective on health/school nursing in England. Promotion of sexual health still encounters resistance, particularly concerning accessibility, acceptance, and the effectiveness of school nursing and education. Revealing significant differences in service and highlighting the need for youth involvement. The findings emphasize the crucial role of school nurses in promoting positive sexual health among youth, advocating for greater visibility of school nurses and clarity in what they offer.	1, 2, 4
Ioannou et al. (2014)	Sexuality education as a collective responsibility: A New health education curriculum in Cyprus	Cyprus	Literature review	School-aged students	The literature review emphasizes a health-promoting perspective on sexuality education within the new health education curriculum in Cyprus. This curriculum underscores the collective responsibility for improving sexual health, moving beyond just individual risk reduction. Training and support for teachers are needed to ensure effective implementation of the curriculum.	1, 2, 4
Brewin et al. (2013)	Behind Closed Doors: School Nurses and Sexual Education	USA	Descriptive semi-structured qualitative interviews	*N* = 18 nurses (full-time equivalent) from 12 high schools	The study explores the role and challenges faced by school nurses in delivering sexuality education in schools. Key barriers include insufficient support from school administration and concerned parents, and a lack of formal education in sexual health. The findings indicate that sexuality education is often informally provided due to limited collaboration. Effective delivery requires appropriate training, a supportive learning environment, and educators who are comfortable addressing the topic. Despite the significant need, school nurses are underutilized, and teacher training often focuses more on crisis management than preventive measures. Collaborative efforts involving school nurses, administrators, teachers, parents, and students are essential for promoting positive sexual health development among students.	1, 2, 3, 4
Chou et al. (2020)	Effectiveness of a school-based social marketing intervention to promote adolescent sexual health	Taiwan	Quasi-experimental design including: One-group online survey, pre- and post-intervention. Interview with 20 adolescents Questionnaires and literature review.	*N* = 1407 students from 24 junior high schools	The study investigates the impact of the intervention program “Starting from Love – Go! Go! Go!.” Findings reveal a notable increase in sexual knowledge and positive shifts in attitudes among participants. There is an association between heightened knowledge levels and more positive outlooks toward sexuality. The program promotes healthier sexual behaviors among students and enhances the efficacy of sexuality education. Student engagement and teacher training are identified as critical factors for improving educational outcomes in this context.	1, 3, 5
Silivri et al. (2021)	Conversations About Sexual and Reproductive Health and Rights – From a School Nurse Perspective	Sweden	Inductive qualitative study design. Individual semi-structured interviews. Inductive qualitative content analysis.	*N* = 14 school nurses Medium to large cities	The article explores the experiences of school nurses in engaging youth in discussions around sexual and reproductive health and rights (SRHR). Key attributes for effective communication include an open-minded demeanor, a sense of curiosity, and a commitment by the school nurse to stay informed about the subject matter. Cultivating neutral and non-judgmental attitudes is crucial for fostering trust, while ensuring that conversations are voluntary. The study highlights the importance of involving youth in various SRHR topics, collaborative efforts with the school, and utilizing resources such as national guidelines as essential components for meaningful discourse and education on SRHR matters.	2, 3, 5
Versloot-Swildens et al. (2023)	Effectiveness of a Comprehensive School-Based Sex Education Program for Young Adolescents in the Netherlands	Netherland	Experimental study. Cluster-randomized controlled trial w. post-test and 6-month follow-up.	*N* = 693 students 63 school classes	“Love is…” is a school-based program aimed at promoting positive sexuality. This article evaluates the program's effectiveness, revealing increased knowledge, attitude changes, and enhanced communication skills among participants. The intervention group showed heightened knowledge compared to the control group, with sustained increases observed six months post-intervention. However, there were no observed changes in attitudes toward online bullying and relationship violence. Participants felt more comfortable discussing sexuality, though their perceived confidence levels remained unchanged. For long-term effectiveness beyond knowledge acquisition, sustained emphasis on positive sexuality, attitudes, communication, and periodic booster sessions are deemed necessary.	3, 5
Wetzel and Sanchez (2023)	What´s Something Yoúve Heard About Sex, But Are Unsure If It´s True? Assessing Middle and High School Students’ Education Questions	USA	Qualitative research design, longitudinal, inductive approach.	*N *= 1335 students from middle and high schools	Adolescents’ sexual experiences often diverge from the content covered in classroom-based sexuality education. Adolescents express a desire for expanded topics, including greater emphasis on positive sexuality and the enjoyment associated with the subject matter. Findings suggest a need for an increased focus on diversity and comprehensive sexuality education from the adolescents’ perspective. Gender disparities were noted, with females more frequently referencing experiences of discomfort or pain, while males tended to discuss pleasure. These findings underscore the imperative to align sexuality education with the perspectives and participation of adolescents.	1, 3, 5
Goldfarb and Lieberman (2021)	Three Decades of Research: The Case of Comprehensive Sexual Education	USA	Systematic literature review.	*N* = 24,579 students Preschool to 12th grade	This study investigates the impact of Comprehensive Sexuality Education (CSE) in schools, with an emphasis on outcomes beyond the prevention of unwanted pregnancy and sexually transmitted infections (STIs). The literature review reveals positive effects of CSE, including enhanced comprehension of sexual diversity, mitigation of relationship violence, improved socio-emotional learning, enhanced mental well-being, promotion of healthier relationships, and cultivation of positive attitudes and values. The study emphasizes the necessity for early and sustained education, alongside novel approaches to sexual health. It also highlights the importance of integrating additional positive themes, such as pleasure and desire, into the curriculum.	3, 4, 5
Pinandari et al. (2023)	Short-Term Effects of a School-Based Comprehensive Sexuality Education Intervention Among Very Young Adolescents in Three Urban Indonesian Settings: A Quasi-Experimental Study	Indonesia	Quasi-experimental study. Two-year intervention: school-based, teacher-led CSE education. Initially school-based but transitioned to online instruction due to COVID-related school closures.	*N* = 3335 18 schools in three urban locations with varying socioeconomic backgrounds	The article investigates the effects of Comprehensive Sexual Education (CSE), revealing increased knowledge, more egalitarian attitudes, and improved communication regarding sexual health, but no effect on personal sexual well-being. A gender difference is observed, with females showing more significant improvements. This highlights the need to address boys’ needs to foster more equitable curricula in CSE. The study underscores the positive impacts of uniform and comprehensive sexual education in schools.	3, 4, 5
Mkumbo (2014)	Students ‘attitudes towards school-based sex and relationships education in Tanzania	Tanzania	Cross-sectional survey. Questionnaire: New Brunswick Students’ Ideas about Sexual Health Education, with modifications.	*N* = 715 students Two districts in Tanzania	The study focused on students’ attitudes toward sexual health education in Tanzanian schools. Results revealed that over 80% of the students expressed a desire for sexual education within the school curriculum, with many advocating for early instruction and coverage of a wide range of topics. However, controversial subjects such as homosexuality and masturbation were generally opposed by most students. Additionally, there was a preference for age-appropriate content. These findings underscore the necessity for culturally tailored and enhanced sexual education programs in Tanzania.	3, 4, 5
Lepkowska (2016)	A greater investment in school nursing is vital to meet needs	England	Commentary in British Journal of School Nursing		The commentary highlights the importance of school nurses in promoting well-being and good health among students. Challenges such as societal pressures underscore the need for comprehensive education on sexual health. The absence of school nurses has significant repercussions in addressing young people's health issues, and the author argues that the government must prioritize ensuring an adequate healthcare workforce in schools.	2, 3
Feuchtwang (2016)	Are we preparing pupils for puberty early enough?	England	Commentary in British Journal of School Nursing		The author points out that adolescents often feel they learn too little and too late about puberty and adolescence. While government guidelines emphasize the importance of learning about puberty before it is experienced, reality shows that this does not always occur. Effective sexual education should encompass knowledge about the body, relationships, emotions, and safety, and it should be tailored to meet the needs of both boys and girls.	3, 5
Ericksen and Weed (2023)	“Three Decades of Research”: A New Sex Ed Agenda and the Veneer of Science	USA	Systematic literature review.	*N* = 24,579 Preschool to 12th grade.	The authors critique Goldfarb and Lieberman's research review of Comprehensive Sexual Education (CSE), pointing out that most studies cited as supportive evidence for CSE do not actually evaluate CSE programs. Additionally, the studies that do assess CSE programs often fail to meet scientific standards. Only one out of 88 studies offers credible evidence for the effectiveness of CSE, and even that evidence is weak. The authors argue that instead of endorsing CSE, Goldfarb and Lieberman are proposing a new type of sexual education without sufficient scientific support.	3, 5
Harris et al. (2022)	Relevant, relatable and reliable: rural adolescents’ sex education preferences	USA	Semi-structured focus group interviews Digital surveys	*N* = 56 students in 11 focus groups School in Indiana	Participants expressed a preference for Comprehensive Sexual Education (CSE) over the abstinence-only approach, feeling that sexual education was either irrelevant or inadequately tailored to their lives. To ensure that sexual education is relevant, relatable, and reliable, it is essential to incorporate youths’ perspectives in the development of the curriculum.	1, 3
Seiler-Ramadas et al. (2021)	Applying Emotional Literacy in CSE for Young People	Australia	Non-systematic approach for narrative reviews		To assist children and adolescents in regulating emotions and behaviors related to sexuality and relationships, as well as to promote mental resilience, self-confidence, and responsible decision-making, the study suggests that emotional literacy practices should be integrated into sexual education. Early initiation of instruction and booster sessions are deemed necessary, and instruction should be comprehensive.	2, 3, 4, 5
Corcoran et al. (2019)	What do we really know about adolescent Sexual Health Education: A Dimensional Concept Analysis	USA	Literature Analysis: Dimensional Examination	Adolescent Sexual Education	This study employs a dimensional analysis approach to explore five dimensions of sexual education for adolescents, aiming to enhance adolescent sexual health. These five dimensions—content, duration of intervention/education, timing, delivery, and measurable outcomes—provide a framework for comparing various interventions/education programs. The absence of a standard for sexual education complicates this task; however, the study suggests that a uniform and comprehensive standard could potentially be developed based on these five dimensions.	3

**Table 3.** Overview of the Included Literature and Coding (*N* = 17).

Studies	Youth participation (1)	The role of educators (2)	Sexual education frameworks (3)	Shift in perspectives on sexuality (4)	Expanding teaching topics within sexual health (5)
Aranda et al. (2018)	X			X	
Acharya et al. (2018)	X	X	X		X
Brewin et al. (2013)	X	X	X		
Chou et al. (2020)	X		X		X
Ericksen and Weed (2023)	X				
Corcoran et al. (2019)			X		
Feuchtwang (2016)			X		X
Goldfarb and Lieberman (2021)			X	X	X
Harris et al. (2022)	X		X	X	
Ioannou et al. (2014)	X	X	X	X	
Lepkowska (2016)		X	X		
Mkumbo (2014)			X	X	X
Pinandari et al. (2023)			X	X	X
Seiler-Ramadas et al. (2021)	X	X	X	X	X
Silivri et al. (2021)		X	X		X
Versloot-Swildens et al. (2023)			X		X
Wetzel and Sanchez (2023)	X		X		X

### Youth Participation in Sexual Education

Aranda et al. (2018) found a significant discrepancy between current sexual education in schools and the actual needs and preferences of young people (Aranda et al., 2018). Several studies emphasize the importance of youth participation and active involvement in the design of curriculum and teaching materials, as crucial for promoting positive sexuality and health cultures (Aranda et al., 2018; Brewin et al., 2013; Ericksen & Weed, 2023; Harris et al., 2022; Ioannou et al., 2014; Seiler-Ramadas et al., 2021). Ioannou et al. (2014) conducted a literature review that highlights a shift from viewing youth as risk-takers to seeing them as actively participating problem solvers in society (Ioannou et al., 2014). Involvement of children and young people in the development of teaching materials, either beforehand or during the process, has shown positive results in terms of increased sexual knowledge and addressing the actual questions they have (Acharya et al., 2018; Chou et al., 2020; Wetzel & Sanchez, 2023). Adolescents desire involvement in the development of education, and research shows that adolescent participation leads to increased learning and knowledge retention (Acharya et al., 2018; Aranda et al., 2018; Brewin et al., 2013; Chou et al., 2020; Harris et al., 2022; Ioannou et al., 2014; Seiler-Ramadas et al., 2021).

### The Role of Educators in Sexuality Education

The role of educators in sex education is multifaceted and crucial for ensuring that young people receive adequate and accurate information. Findings suggest that school nurses possess more knowledge about sexual health, teach broader topics, and operate under different structures than teachers (Brewin et al., 2013). Research indicates that well-educated educators are required to promote attitude change and positive sexuality (Acharya et al., 2018; Aranda et al., 2018; Brewin et al., 2013; Ioannou et al., 2014; Lepkowska, 2016; Seiler-Ramadas et al., 2021). Educators should possess extensive competence in sexual health, be comfortable in the role, be motivated, be aware of their own biases related to sexuality, and be able to establish trust (Brewin et al., 2013; Silivri et al., 2021). In Nepal, school students often face difficulties in asking questions about sexual health due to teachers’ lack of knowledge and limited teaching techniques (Acharya et al., 2018). Aranda and colleagues found that educators initiating discussions about emotions and relationships overcame students’ shyness (Aranda et al., 2018).

Involving young people in developing and implementing sexuality education programs ensures relevance and engagement. Seiler-Ramadas and colleagues (2021) argue that educators benefit from using more informal and participatory teaching methods to engage students and create an environment where questions about sexual and reproductive health (SRH) can be discussed openly (Seiler-Ramadas et al., 2021). Participatory methods such as group discussions, role-playing, and quizzes can contribute to an interactive and educational experience and are more used by school nurses than teachers (Chou et al., 2020; Goldfarb & Lieberman, 2021; Ioannou et al., 2014; Silivri et al., 2021). In the Cypriot health education model, a collective approach is emphasized, aiming to develop children and young people's potential as active and responsible citizens (Ioannou et al., 2014). It is important to consider how sexual health services and information offerings can be designed to be easily accessible and user-friendly (Lepkowska, 2016). Schools can also facilitate collaboration with parents and the community to build trustful relationships, identify students’ needs, and improve communication with parents (Brewin et al., 2013). School leadership should encourage open dialogue around the topic (Silivri et al., 2021).

### Challenges and Recommendations for Standardizing Sexual Education Frameworks

The literature indicates a lack of a standard for sexual education, leading to challenges in assessing the effectiveness of the provided sexual education (Corcoran et al., 2019; Ioannou et al., 2014; Lepkowska, 2016; Pinandari et al., 2023; Silivri et al., 2021). In several European countries, sexual health is not a separate subject in the curriculum; instead, each school is responsible for determining its implementation (Ioannou et al., 2014). Several of the studies suggest that the curriculum should include research-based, relevant, relatable, and reliable information that is updated, accessible to all, and tailored to the local context (Acharya et al., 2018; Harris et al., 2022; Seiler-Ramadas et al., 2021). For planning and implementation of sexual health education, systematic collaboration between school nurses and schools is recommended (Silivri et al., 2021). Brewin et al. (2013) found that school nurses often lack awareness of the competency goals in the curriculum and are excluded from school activities (Brewin et al., 2013). The literature suggests various teaching methods and tools create a conducive learning environment (Chou et al., 2020; Goldfarb & Lieberman, 2021). Early, age-appropriate, as well as education of extended duration are relevant factors for effective frameworks (Feuchtwang, 2016; Goldfarb & Lieberman, 2021; Mkumbo, 2014). Two studies highlight gender differences in education (Pinandari et al., 2023; Wetzel & Sanchez, 2023), and the literature emphasizes the importance of the curriculum meeting the needs of both boys and girls (Feuchtwang, 2016).

Time and booster sessions emerge as crucial factors for building trust and fostering attitudinal changes regarding sexuality (Goldfarb & Lieberman, 2021; Pinandari et al., 2023; Seiler-Ramadas et al., 2021; Silivri et al., 2021; Versloot-Swildens et al., 2023). One study suggested that shifting from traditional sexual education to more emotionally rich and comprehensive education in all schools is necessary to achieve the intended effects outlined in international guidelines (Seiler-Ramadas et al., 2021).

### The Need for a Conceptual Shift in Perspectives on Sexuality in Education

Teaching sexual health proves to be challenging and requires a theoretical and conceptual shift (Aranda et al., 2018). To address the need, Pinandari and colleagues (2023) argue that sexual education should focus on promoting health and well-being rather than merely preventing problems. Education must consider the multitude of factors influencing sexuality, including biological, psychological, social, economic, political, ethical, legal, historical, religious, and spiritual factors (Ioannou et al., 2014). Overall, curricula often lack health-promoting perspectives in sexual education, with the primary focus on biological and preventive factors (Goldfarb & Lieberman, 2021; Ioannou et al., 2014; Pinandari et al., 2023). Sexuality is seen as a resource for health and quality of life, and it is important to understand the interplay of numerous factors that influence sexuality (Harris et al., 2022, Ioannou et al., 2014). Students should be regarded as problem solvers rather than at-risk individuals needing protection. This involves providing them with the knowledge and skills to make informed decisions and promote healthy choices within their communities (Ioannou et al., 2014). Goldfarb and Lieberman (2021) argue that sexuality should not be taught in isolation but integrated into other subjects such as health, social studies, literature, and the arts (Goldfarb & Lieberman, 2021). Such integration can provide a more holistic understanding of sexuality's role in human life (Ioannou et al., 2014). Also, inadequate emotional functioning can harm sexual health and is connected to other public health issues, such as violence and substance abuse (Mkumbo, 2014; Seiler-Ramadas et al., 2021).

### Expanding Teaching Topics in Sexual Health Education: A Call for a Positive Focus

The included literature points out that the focus of sexual education is directed towards preventive and biological aspects, while there is a need for a sex-positive focus. Research shows this leads to increased sexual competence and positive attitudes towards sexual health (Acharya et al., 2018; Chou et al., 2020; Pinandari et al., 2023; Versloot-Swildens et al., 2023). The literature indicates that there are many topics not covered in current sexual education, including pleasure, desire, and positive sexuality (Chou et al., 2020; Goldfarb & Lieberman, 2021; Pinandari et al., 2023; Seiler-Ramadas et al., 2023; Silivri et al., 2021; Wetzel & Sanchez, 2023). Topics desired by children and young people in education cover both preventive and health-promoting factors, including, among others, sexually transmitted infections, HIV/AIDS, puberty and the body, pleasure, love, gender relations and gender identity, online bullying, stereotypes, relationship violence, communication, equality, boundaries, pornography, topics related to self-love, body image, performance anxiety, mechanics of sex, and anal and oral sex (Acharya et al., 2018; Feuchtwang, 2016; Mkumbo, 2014; Silivri et al., 2021; Wetzel & Sanchez, 2023). One study by Mkumbo (2014) indicates that the topics children and young people wish to learn about in sexuality education are influenced by cultural factors. Findings also indicate that school nurses observe that children and young people lack knowledge about sexual health (Silivri et al., 2021).

## Discussion

This scoping review aims to conduct a review of existing research pertaining to sexual education, focusing on the role of school nurses in promoting positive sexual health among children and adolescents through sexual education.

The WHO (2023) definition of sexual education provides a comprehensive framework that emphasizes the importance of addressing both preventive and positive aspects of sexual health, which aligns closely with the findings of this scoping review. Findings from this current scoping review indicate that sexual education is a multifaceted subject that intersects with various aspects of children's and adolescents’ lives, making it crucial to ensure its effectiveness and relevance to their context (Aranda et al., 2018; UNESCO et al., 2018). While the prevention of risk-taking sexual behavior has been a central focus, there is an increased desire to promote positive sexual health and enhance young people's understanding of how sexuality and perception of sexuality are influenced and shaped by various factors, including age, gender and social, religious and cultural influence (Acharya et al., 2018; Chou et al., 2020; Ioannou et al., 2014; Mkumbo, 2014; Silivri et al., 2021; WHO, 2023). This desire can be understood within the context of societal perceptions of youth sexuality and the broader concept of sexuality, highlighting the need for a comprehensive shift in the approach to sexual education. Adolescent sexuality remains stigmatized and requires attitude changes, which can be a lengthy process (Goldfarb & Lieberman, 2021; Ioannou et al., 2014). The included studies reveal a gap between the sexual education children and young people desire, which includes positive sexuality, and the current focus of many educational programs (Chou et al., 2020; Goldfarb & Lieberman, 2021; Pinandari et al., 2023; Wetzel & Sanchez, 2023). Addressing this gap requires a shift towards incorporating themes of pleasure, desire, and healthy relationships into the curriculum.

Goldfarb and Lieberman (2021) points out in their discussion that topics such as pleasure and desire are not represented in the curricula reviewed (Goldfarb & Lieberman, 2021). This contrasts with the topics students and school nurses identify as important contrasts with the topics of children and young people, as well as the topics school nurses identify as lacking (Mkumbo, 2014; Silivri et al., 2021).

Several of the included studies highlight the need for children and adolescents’ participation in the design of educational programs to ensure their relevance and effectiveness (Acharya et al., 2018; Brewin et al., 2013; Chou et al., 2020). There is a significant disparity between what children and young people desire and the sexual education they receive (Aranda et al., 2018). This gap is further widened by the vast amount of online information about sexuality, which often contradicts traditional sexual education (Seiler-Ramadas et al., 2021). Addressing young people's curiosity about positive sexuality is crucial for promoting safe sex practices (Seiler-Ramadas et al., 2021). Studies show that involving youth in designing educational content increases their sexual health knowledge and attitudes while answering their genuine questions (Chou et al., 2020; Wetzel & Sanchez, 2023).

In this review, we found research related to the competence and training of educators of sexual health (Aranda et al., 2018; Brewin et al., 2013; Silivri et al., 2021). Both school nurses and teachers are pivotal in delivering effective sexual education. Therefore, continuous professional development and specialized training in sensitive topics and contemporary issues in sexual health are essential to meet the evolving needs of young people (The Norwegian Directorate of Health, 2016). However, the findings suggest that it is crucial and largely up to the educators themselves to keep up to date on topics within sexual health in order to keep pace with children's and adolescents’ knowledge on the subject (Silivri et al., 2021). Our findings point out that educators lack competence and desire more training on sexual health (Goldfarb & Lieberman, 2021). One study indicates that school nurses have a great amount of confidence in their own competence and that they have opportunities for courses and further education (Versloot-Swildens et al., 2023). Seiler-Ramdas and colleagues state that sexual education is often provided by educators without expertise and that this reflects the low status of sexual education (Seiler-Ramadas et al., 2021). Several studies suggest that using various tools such as online programs, role-playing, emotional literacy, and humor in education increases engagement among young people. These tools are more commonly used by school nurses than teachers, contributing to more effective education (Chou et al., 2020; Goldfarb & Lieberman, 2021; Silivri et al., 2021).

UNESCO's (2018) report underlines that sexual education should be more comprehensively integrated into the education system and be anchored in international guidelines and standards (Silivri et al., 2021; UNESCO et al., 2018). Ioannou et al. (2014) point out that in many European countries, sexual education is not a separate subject in the curriculum, and schools have varying practices regarding the inclusion of sexual health in education (Ioannou et al., 2014). Differences in approaches to sexual education across international borders may result in not all children and adolescents receiving the education they are entitled to (Ioannou et al., 2014; UNESCO, 2008; UNESCO et al., 2018). Documents related to sexual education in Europe are rarely translated and published, which makes it difficult to find documents and information in English (Ioannou et al., 2014). Regular updates on sexual education are requested by both teachers and students, and research also shows that consistent education over time promotes positive sexual health and lasting attitude changes (Seiler-Ramadas et al., 2021; UNESCO et al., 2018). One suggestion is to consider the need for a more coordinated approach to sexual education to ensure a more comprehensive and effective learning experience for students.

Research on the effectiveness of sexual education programs is challenging when there is no standardized framework to evaluate against (Corcoran et al., 2019; Ericksen & Weed, 2023). UNESCO's guidelines for Comprehensive Sexuality Education provide guidance on relevant topics to be included in sexual education, but the implementation of these guidelines varies and may result in important aspects, such as positive sexuality and pleasure, being overlooked in favor of a focus on risk-taking behavior (Ioannou et al., 2014; Pinandari et al., 2023; Silivri et al., 2021; UNESCO et al., 2018). Also, internationally, the role and mandate of school nurses in sexual education are practiced differently (WHO & UNESCO, 2021). In Norway and Sweden, school health services, including the role of the school nurse, are legally mandated. Despite variations in their specific mandates and implementations, school nurses remain an integral part of the educational system (Forskrift om helsestasjons- og skolehelsetjenesten, 2018; Sveriges riksdag, 2010). This contrasts with countries such as the United States, where the need for such services is determined by the state or individual schools (Planned Parenthood Action Fund, 2025). Our study identifies a lack of research from Nordic countries. To ensure that education reaches all students and is of high quality, more resources with up-to-date knowledge on the subject are required, particularly from countries where the mandate of school nurses is a statutory service (Brewin et al., 2013; Corcoran et al., 2019; Feuchtwang, 2016).

### Strengths and Limitation

A strength of this study lies in the comprehensive search of literature that was conducted. However, the study does have limitations. Notably, the reference lists in the included articles were not reviewed for secondary sources. Additionally, in accordance with the frameworks by Arksey and O’Malley (2005) regarding scoping reviews, a quality assessment of the included studies has been conducted (Arksey & ÓMalley, 2005).

The findings are limited as part of the research question pertains to the role of the school nurse. There is no international standard for the mandate of school nurses. Therefore, the role will vary, and consequently, so will the findings. Considering the absence of an international mandate for school nurses and considering a report by WHO and UNESCO (2021) that highlights the lack of school nurses stationed within schools in several countries, it has been concluded that such a mandate does not exist.

### Relevance to Clinical Practice and Implications for Further Research

This review points to a lack of longitudinal research on sexual education. It demonstrates that promoting positive sexuality requires a shift in attitudes and perceptions toward sexuality, which takes time (Goldfarb & Lieberman, 2021; Ioannou et al., 2014). Research on sexual education often focuses on short-term interventions and educational programs (Chou et al., 2020). The findings in this review indicate a need for an internationally standardized sexual education program, including the role of school nurses, to facilitate further research on the effectiveness of sexual education.

## Conclusion

The findings in this review, through the 17 articles examined, highlight the significant contributions of school nurses in delivering sexual education programs, with youth expressing a preference for external, qualified educators such as school nurses to deliver sexual education. However, despite the crucial role they play, the amount of information specifically about school nurses’ contributions and effectiveness in this context is limited. This highlights a gap in the existing literature and suggests a need for further research to fully understand and optimize the role of school nurses in sexual education.

Our initial screening process revealed extensive research on preventive aspects such as harmful sexual behavior, sexually transmitted infections, and unwanted pregnancies, but found limited literature on promoting positive sexuality. The articles reviewed highlight sexual education as a complex and pivotal component of children and adolescents’ education and health-promoting initiatives. They suggest that a comprehensive approach, active participation, well-trained educators, appropriate frameworks and tools, an open perspective on sexuality, and a wide range of topics are necessary to promote positive sexuality among children and young people. Notably, there is a lack of representation of these topics in the existing literature, particularly from Nordic countries. This emphasizes the need for further, more diverse research on how sexual education can enhance positive sexual health among youth, with a particular focus on the role of school nurses.

In conclusion, while school nurses play a significant role in promoting positive sexual health through education, there is a need for more focused research on their specific contributions and effectiveness. This will help to further enhance sexual health promotion strategies among youth.
